# Human mitochondrial ribosomes can switch structural tRNAs – but when and why?

**DOI:** 10.1080/15476286.2017.1356551

**Published:** 2017-09-13

**Authors:** Zofia Chrzanowska-Lightowlers, Joanna Rorbach, Michal Minczuk

**Affiliations:** a The Wellcome Trust Centre for Mitochondrial Research, Institute of Neuroscience, Newcastle University, Newcastle upon Tyne, England, UK; b Department of Medical Biochemistry and Biophysics, Karolinska Institutet, Retzius väg 8, Stockholm, Sweden; c MRC Mitochondrial Biology Unit, Wellcome Trust/MRC Building, Hills Road, Cambridge, England, UK

**Keywords:** Human, mammalian, Mitochondria, ribosomes, rRNA, tRNA

## Abstract

High resolution cryoEM of mammalian mitoribosomes revealed the unexpected presence of mitochondrially encoded tRNA as a structural component of mitochondrial large ribosomal subunit (mt-LSU). Our previously published data identified that only mitochondrial (mt-) tRNA^Phe^ and mt-tRNA^Val^ can be incorporated into mammalian mt-LSU and within an organism there is no evidence of tissue specific variation. When mt-tRNA^Val^ is limiting, human mitoribosomes can integrate mt-tRNA^Phe^ instead to generate a translationally competent monosome. Here we discuss the possible reasons for and consequences of the observed plasticity of the structural mt-tRNA integration. We also indicate potential direction for further research that could help our understanding of the mechanistic and evolutionary aspects of this unprecedented system.

Protein synthesis is a fundamental cellular process, and its regulation exerts exquisite control over post-transcriptional gene expression. Many aspects of this process and its machinery are conserved from bacteria to higher eukaryotes. The essential components being a mRNA template to translate, and a ribosome to perform this function. The main phases of protein synthesis are initiation, elongation, and termination, followed by ribosome recycling.^1^^,^^2^ Although many elements display a level of conservation, closer inspection reveals distinct differences between systems, particularly with respect to mitochondria. For example, the mRNA template in many systems has a non-coded 3′-extension, namely the poly(A) tail. In bacteria, this targets the transcript for degradation, while for transcripts in the eukaryotic cytosol, this tail is generally coated in poly(A)-binding proteins that protect and enhance RNA stability.^3^^,^^4^ Poly(A) tails are also present on mitochondrially-encoded transcripts in plant organelles, where the presence of a poly(A) tail follows the bacterial paradigm.^5^^,^^6^ Such tails are also present on mitochondrially-encoded transcripts in trypanosomes, where tails can be present as short oligo(A) extensions or longer forms. The length is dependent on the editing status, which in turn dictates degradation or stability.^7^ Yeast mitochondria eschew this form of regulation and have no poly(A) tail.^8^ The function of the poly(A) tail in mammalian mitochondria, therefore, remains an enigma, with no obvious universal function. The one role that is clear is that they are needed to complete the UAA at the end of 7 out of 13 open reading frames, thereby generating complete stop codons. Research from several groups using different approaches has shown that the poly(A) tail is important and has variable functions, including increasing or decreasing the stability of specific transcripts.^9-14^

Other features of the mRNA template also differ between systems, but the greatest differences have been described for the translation machinery – namely, the ribosome. The basic composition of this complex apparatus is two multi-protein subunits of different sizes, with rRNA components in both the small (SSU) and large (LSU) subunits. This template is true for the majority of ribosomes, including mitoribosomes. Every rule has an exception, and the mammalian mitoribosome seems to be proving the exception in more ways than one. Early on it was discovered to have novel protein components, as well as a truncated RNA species.^15-17^ For both the mitochondrial (mt-) SSU and mt-LSU almost 50% of the polypeptides were shown to be mitochondrially specific and lack bacterial orthologues. Furthermore, many of the proteins with bacterial orthologues have N and/or C-terminal extensions that are mitochondrially specific. The rRNA species within mammalian mitoribosomes were believed to be restricted to one per subunit. In each case, the elimination of specific regions, while retaining crucial functional domains, has rendered rRNAs shorter than their bacterial/eukaryotic cytosolic counterparts. The combination of more proteins and shorter rRNAs has generated a mitoribosome with a protein to RNA ratio of 70:30, which is essentially reversed compared with other ribosomes that have 70% rRNA and only 30% protein. Although, the rRNA species within mammalian mitoribosomes were generally believed to be restricted to one per subunit, published data exist on mitochondrial matrix localization of cytosolic 5S rRNA (5S rRNA), with a pool of 5S rRNA being proposed to be associated with the mitochondrial ribosomes.^18^

Significant advances in cryoEM technology have brought about a revolution in structural biology, generating high resolution data of large complexes. This has revealed yet more idiosyncrasies of the mammalian mitoribosome.^19^^,^^20^ The long-held belief that only two mitochondrially-encoded RNA species were present was shattered as a third RNA species originated from the mitochondrial genome was identified.^21^^,^^22^ This finding concurrently resolved the debate of whether cytosolic 5S rRNA could be imported and integrated into the mt-LSU. This unexpected RNA species was found to occupy the position in which the 5S RNA would be found in the bacterial LSU. The reason for substitution of 5S rRNA for a mitochondrially-encoded tRNA in the mt-LSU is unclear, but could stem from the inability of the mammalian organelle to efficiently import RNA from the cytoplasm (see discussion in^23^). CryoEM and sequencing data pertaining to the human mitoribosome was generated by Ramakrishnan and colleagues, confirming the RNA species to be mt-tRNA^Val^^22^ Data from porcine mitoribosomes generated by Ban and coworkers conflicted with this, describing mt-tRNA^Phe^ to be the newly identified component.^21^ Thus, the question arose as to whether different mammals incorporate different mt-tRNAs, or whether the selection is restricted to only mt-tRNA^Phe^ or mt-tRNA^Val^?

Our collaborative endeavors have explored this question by analyzing mitochondrial lysates from human cells and using isokinetic sucrose gradients to investigate the RNA species associated with the mt-LSU. They confirmed that human mitoribosomes integrate mt-tRNA^Val^, while porcine particles favor mt-tRNA^Phe^, with no evidence of integration of other mt-tRNAs. By analyzing mitochondria from other mammals, including cows, rats and macaques, we confirmed that only mt-tRNA^Phe^ or mt-tRNA^Val^ are selected. Initial data was collected from a single tissue from each organism, therefore, it was possible that the selection of mt-tRNA^Phe^ or mt-tRNA^Val^ was a consequence of tissue-specific variation. To address this question, a selection of different tissues from several mammals was analyzed in a similar fashion. This confirmed that the mt-tRNA selected to be integrated into the mt-LSU within a species was the same, regardless of tissue.^24^ So, what drives mt-tRNA selection? Porcine data, in particular, suggested a favoring of mt-tRNA^Phe^ but not a complete exclusion of mt-tRNA^Val^. Does this suggest that these 2 tRNA species are interchangeable?

A long-standing impediment to investigating mammalian mitochondrial gene expression is the refractory nature of the organelle to genetic transformation.^25^ To date, the introduction of genetic reporters of any kind has not been documented in a reproducible and efficient manner - limiting possible approaches to address such questions. Such research has had to rely, therefore, on naturally-occurring mutations identified in patients presenting with mitochondrial disease. One such mutation has been described within the gene encoding mt-tRNA^Val^. Early investigations determined that the mutation destabilised mt-tRNA^Val^, thereby reducing steady-state levels in tissues and to a lesser extent in cell lines.^26^ Clonal cybrid cell lines were derived that harboured all mutant copies of the mitochondrial genome (homoplasmy) and recapitulated the reduced levels of mt-tRNA^Val^ and demonstrated mildly reduced intra-mitochondrial translation by both metabolic labeling of *de novo* synthesis and by steady-state levels of mitochondrially encoded COXII protein.^27^^,^^28^ In light of the cryo-EM-defined structural integration of mt-tRNA^Val^, this cell line was re-investigated. Remarkably, sufficient plasticity had been retained to allow incorporation of mt-tRNA^Phe^ into the mt-LSU. Perhaps more surprisingly, even when steady-state levels of mt-tRNA^Val^ were elevated (through overexpression of the cognate valyl tRNA synthetase), mt-tRNA^Phe^ remained as the structural component integrated into the mt-LSU. This strongly suggested that the difference in structure of the mt-tRNA^Val^ caused by the mutation was sufficient for it to be recognized as aberrant and rejected by the large subunit, while discerning that the structure of mt-tRNA^Phe^ was acceptable.^24^

Although these observations further our understanding of the mammalian translation machinery, they also evoke more questions. Why are only mt-tRNA^Phe^ and mt-tRNA^Val^ used? One possibility is that the tertiary structure of these 2 RNA species permits their integration, and that bulkier mt-tRNAs - or those lacking the D-arm - do not provide an appropriate scaffold for large subunit formation.^29^ Our current databases do not have sufficient information on mammalian mt-tRNAs to model whether the structural features render only mt-tRNA^Phe^ or mt-tRNA^Val^ compatible, and why the shape of the mutant mt-tRNA^Val^ makes it less favored than the wild type mt-tRNA^Phe^. If it is a structural rationale that excludes the integration of all candidates except mt-tRNA^Phe^ and mt-tRNA^Val^, it would strengthen the case for selection of one or other of these two mt-tRNAs being a consequence of their position in the mitochondrial genome. For ribosomes that incorporate a 5S rRNA species, the gene is frequently found in the same transcription unit as the major rRNA species. This arrangement is somewhat recapitulated in the mammalian mitochondrial genome, where the polycistronic transcript generated from the heavy strand promoter begins with mt-tRNA^Phe^, and sandwiches mt-tRNA^Val^ between the 12S and 16S rRNAs.^30^ There are reports that transcription can terminate a few nucleotides beyond the 16S gene, extending into mt-tRNA^Leu^, while other transcription events continue through to generate longer polycistronic transcripts encompassing almost the entire heavy strand sequence.^31^ Whether or not the shorter rRNA-containing polycistron is generated at a higher frequency does not alter the co-localization of these two mt-tRNA species. Such genomic organization would mean that processing of the polycistron would release both mt-rRNAs and these two mt-tRNAs in spatial proximity, making them immediately accessible for co-integration with rRNA into the large subunit. This gene arrangement is highly conserved in mammals, but not in all Metazoa (Fig. 1). Many invertebrates have widely separated mt-rRNA genes that lack both non-coding and/or potential promoter sequences immediately upstream of these genes. This raises the question of how their mitoribosome assembly is spatially organized and which, if any, mt-tRNA is incorporated into the LSU. Should a high resolution structure of such mitoribosomes become available, it will undoubtedly begin to answer some of the questions.

**Figure 1.**
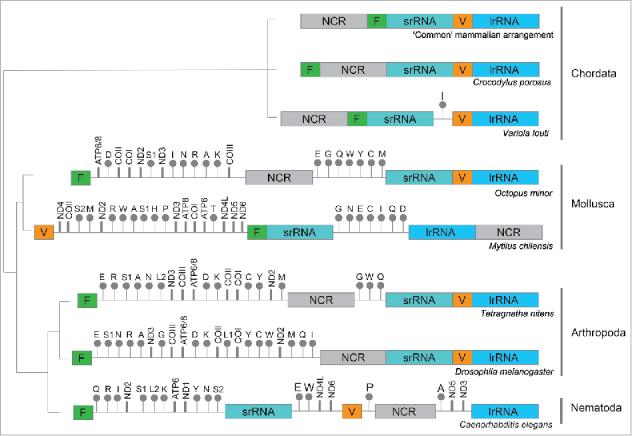
*Gene arrangement of rRNAs from various metazoan phyla* Schematic maps indicating the relative position of the genes encoding the mt-rRNA, mt-tRNA^Val^, and mt-tRNA^Phe^ from various phyla are presented by evolutionary origin. The positions of various mt-tRNAs genes (designated by their single letter code), highlighting those encoding mt-tRNA^Val^ (orange) and mt-tRNA^Phe^ (green), are shown relative to the rRNA genes (srRNA, lrRNA designate RNA for small or large subunit respectively). Intervening non-coding (NCR) regions are indicated, as are genes for protein coding genes.^35-40^

If, at least in mammals, it is the proximity of the mt-tRNA^Phe^ and mt-tRNA^Val^ to the rRNA genes that gives the temporal as well as spatial availability during mitoribosome biogenesis, then how does the process discriminate between mt-tRNA^Phe^ and mt-tRNA^Val^, as is clearly the case between bovine and human, for example. Do different mammals post-transcriptionally modify these 2 mt-tRNAs differently to direct selectivity? Could any such modification impart a change in charge, or in conformation of the mt-tRNA that makes their incorporation more or less likely? These are certainly avenues that are waiting to be explored. It may be that a proportion of mt-tRNA escapes modification as it is incorporated into the large subunit, or perhaps mt-tRNAs are integrated into the nascent large subunit before processing occurs. In the eukaryotic cytosol, newly-synthesized 5S rRNA is complexed with the ribosomal protein L5 (uL18) and other RNA-binding proteins for its incorporation into the ribosome.^32^^,^^33^ Similarly, uL18 is essential for 5S incorporation into bacterial LSU.^34^ Do similar tRNA-protein complexes exist in mitochondria? Were this to be the case, the proteins involved in this interaction could dictate selectivity of the incorporated tRNA species. As no uL18 homolog exists in mammalian mitochondria, other factors, potentially certain mitochondria-specific proteins, might be involved. A better understanding of the timing of the events taking place in the RNA granules, where the mitoribosome biogenesis initiates may shed light on this complex process.

## References

[1] Lightowlers RN, Rozanska A, Chrzanowska-Lightowlers ZM Mitochondrial protein synthesis: figuring the fundamentals, complexities and complications, of mammalian mitochondrial translation. FEBS Lett. 2014;588:2496–503. doi:10.1016/j.febslet.2014.05.054. 24911204 PMC4099522

[2] Ott M, Amunts A, Brown A Organization and Regulation of Mitochondrial Protein Synthesis. Ann Rev Biochem. 2016;85:77–101. doi:10.1146/annurev-biochem-060815-014334. 26789594

[3] O'Hara EB, Chekanova JA, Ingle CA, Kushner ZR, Peters E, Kushner SR Polyadenylylation helps regulate mRNA decay in Escherichia coli. Proc Natl Acad Sci U S A. 1995;92:1807–11. doi:10.1073/pnas.92.6.1807. 7534403 PMC42371

[4] Sarkar N Polyadenylation of mRNA in prokaryotes. Annu Rev Biochem. 1997;66:173–97. https://doi.org/10.1146/annurev.biochem.66.1.173. 9242905

[5] Kudla J, Hayes R, Gruissem W Polyadenylation accelerates degradation of chloroplast mRNA. EMBO J. 1996;15:7137–46. 9003789 PMC452540

[6] Lisitsky I, Klaff P, Schuster G Addition of destabilizing poly (A)-rich sequences to endonuclease cleavage sites during the degradation of chloroplast mRNA. Proc Natl Acad Sci U S A. 1996;93:13398–13403. doi:10.1073/pnas.93.23.13398. 8917603 PMC24105

[7] Kao CY, Read LK Opposing effects of polyadenylation on the stability of edited and unedited mitochondrial RNAs in Trypanosoma brucei. Mol Cell Biol. 2005;25:1634–1644. https://doi.org/10.1128/MCB.25.5.1634-1644.2005. 15713623 PMC549368

[8] Groot GS, Flavell RA, Van Ommen GJ, Grivell LA Yeast mitochondrial RNA does not contain poly(A). Nature. 1974;252:167–169. doi:10.1038/252167a0. 4424381

[9] Slomovic S, Laufer D, Geiger D, Schuster G Polyadenylation and degradation of human mitochondrial RNA: the prokaryotic past leaves its mark. Mol Cell Biol. 2005;25:6427–35. https://doi.org/10.1128/MCB.25.15.6427-6435.2005. 16024781 PMC1190340

[10] Wydro M, Bobrowicz A, Temperley RJ, Lightowlers RN, Chrzanowska-Lightowlers ZM Targeting of the cytosolic poly(A) binding protein PABPC1 to mitochondria causes mitochondrial translation inhibition. Nucleic Acids Res. 2010;38:3732–42. https://doi.org/10.1093/nar/gkq068. 20144953 PMC2887948

[11] Rorbach J, Minczuk M The post-transcriptional life of mammalian mitochondrial RNA. Biochem J. 2012;444:357–373. doi:10.1042/BJ20112208. 22642575

[12] Rorbach J, Bobrowicz A, Pearce S, Minczuk M Polyadenylation in bacteria and organelles. Methods Mol Biol. 2014;1125:211–27. 24590792 10.1007/978-1-62703-971-0_18

[13] Rorbach J, Nicholls TJ, Minczuk M PDE12 removes mitochondrial RNA poly(A) tails and controls translation in human mitochondria. Nucleic Acids Res. 2011;39:7750–7763. doi:10.1093/nar/gkr470. 21666256 PMC3177208

[14] Wilson WC, Hornig-Do HT, Bruni F, Chang JH, Jourdain AA, Martinou JC, Falkenberg M, Spahr H, Larsson NG, Lewis RJ, et al. A human mitochondrial poly(A) polymerase mutation reveals the complexities of post-transcriptional mitochondrial gene expression. Human Molecular Genetics. 2014;23:6345–55. doi:10.1093/hmg/ddu352. 25008111 PMC4222368

[15] O'Brien TW Properties of human mitochondrial ribosomes. IUBMB Life. 2003;55:505–13. doi:10.1080/15216540310001626610. 14658756

[16] Sharma MR, Booth TM, Simpson L, Maslov DA, Agrawal RK Structure of a mitochondrial ribosome with minimal RNA. Proc Natl Acad Sci U S A. 2009;106:9637–42. doi:10.1073/pnas.0901631106. 19497863 PMC2700991

[17] Sharma MR, Koc EC, Datta PP, Booth TM, Spremulli LL, Agrawal RK Structure of the mammalian mitochondrial ribosome reveals an expanded functional role for its component proteins. Cell. 2003;115:97–108. doi:10.1016/S0092-8674(03)00762-1. 14532006

[18] Smirnov A, Entelis N, Martin RP, Tarassov I Biological significance of 5S rRNA import into human mitochondria: role of ribosomal protein MRP-L18. Genes Dev. 2011;25:1289–305. doi:10.1101/gad.624711. 21685364 PMC3127430

[19] Amunts A, Brown A, Toots J, Scheres SHW, Ramakrishnan V Ribosome. The structure of the human mitochondrial ribosome. Science. 2015;348:95–8. doi:10.1126/science.aaa1193. 25838379 PMC4501431

[20] Greber BJ, Bieri P, Leibundgut M, Leitner A, Aebersold R, Boehringer D, Ban N Ribosome. The complete structure of the 55S mammalian mitochondrial ribosome. Science. 2015;348:303–308. 25837512 10.1126/science.aaa3872

[21] Greber BJ, Boehringer D, Leibundgut M, Bieri P, Leitner A, Schmitz N, Aebersold R, Ban N The complete structure of the large subunit of the mammalian mitochondrial ribosome. Nature. 2014;515, 283–6. 25271403 10.1038/nature13895

[22] Brown A, Amunts A, Bai XC, Sugimoto Y, Edwards PC, Murshudov G, Scheres SH, Ramakrishnan V Structure of the large ribosomal subunit from human mitochondria. Science. 2014;346:718–22. doi:10.1126/science.1258026. 25278503 PMC4246062

[23] Gammage PA, Gaude E, Van Haute L, Rebelo-Guiomar P, Jackson CB, Rorbach J, Pekalski ML, Robinson AJ, Charpentier M, Concordet JP, et al. Near-complete elimination of mutant mtDNA by iterative or dynamic dose-controlled treatment with mtZFNs. Nucleic Acids Res. 2016;44:7804–16. doi:10.1093/nar/gkw676. 27466392 PMC5027515

[24] Rorbach J, Gao F, Powell CA, D'Souza A, Lightowlers RN, Minczuk M, Chrzanowska-Lightowlers ZM Human mitochondrial ribosomes can switch their structural RNA composition. Proc Natl Acad Sci U S A. 2016;113:12198–201. doi:10.1073/pnas.1609338113. 27729525 PMC5087001

[25] Lightowlers RN Mitochondrial transformation: time for concerted action. EMBO Rep. 2011;12:480–1. doi:10.1038/embor.2011.93. 21629303 PMC3128293

[26] McFarland R, Clark KM, Morris AA, Taylor RW, Macphail S, Lightowlers RN, Turnbull DM Multiple neonatal deaths due to a homoplasmic mitochondrial DNA mutation. Nat Genet. 2002;30:145–46. doi:10.1038/ng819. 11799391

[27] Rorbach J, Yusoff AA, Tuppen H, Abg-Kamaludin DP, Chrzanowska-Lightowlers ZM, Taylor RW, Turnbull DM, McFarland R, Lightowlers RN Overexpression of human mitochondrial valyl tRNA synthetase can partially restore levels of cognate mt-tRNAVal carrying the pathogenic C25U mutation. Nucleic Acids Res. 2008;36:3065–74. doi:10.1093/nar/gkn147. 18400783 PMC2396425

[28] Hornig-Do HT, Montanari A, Rozanska A, Tuppen HA, Almalki AA, Abg-Kamaludin DP, Frontali L, Francisci S, Lightowlers RN, Chrzanowska-Lightowlers ZM Human mitochondrial leucyl tRNA synthetase can suppress non cognate pathogenic mt-tRNA mutations. EMBO Mol Med. 2014;6:183–93. 24413189 10.1002/emmm.201303202 PMC3927954

[29] Suzuki T, Nagao A, Suzuki T Human mitochondrial tRNAs: biogenesis, function, structural aspects, and diseases. Annu Rev Genet. 2011;45:299–329. doi:10.1146/annurev-genet-110410-132531. 21910628

[30] Anderson S, Bankier AT, Barrell BG, de Bruijn MH, Coulson AR, Drouin J, Eperon IC, Nierlich DP, Roe BA, Sanger F, et al. Sequence and organization of the human mitochondrial genome. Nature. 1981;290:457–65. doi:10.1038/290457a0. 7219534

[31] Gustafsson CM, Falkenberg M, Larsson NG Maintenance and Expression of Mammalian Mitochondrial DNA. Annu Rev Biochem. 2016;85:133–60. doi:10.1146/annurev-biochem-060815-014402. 27023847

[32] Steitz JA, Berg C, Hendrick JP, La Branche-Chabot H, Metspalu A, Rinke J, Yario T A 5S rRNA/L5 complex is a precursor to ribosome assembly in mammalian cells. J Cell Biol. 1988;106:545–56. doi:10.1083/jcb.106.3.545. 3279045 PMC2115095

[33] Zhang J, Harnpicharnchai P, Jakovljevic J, Tang L, Guo Y, Oeffinger M, Rout MP, Hiley SL, Hughes T, Woolford JL Jr. Assembly factors Rpf2 and Rrs1 recruit 5S rRNA and ribosomal proteins rpL5 and rpL11 into nascent ribosomes. Genes Dev. 2007;21:2580–92. doi:10.1101/gad.1569307. 17938242 PMC2000323

[34] Rohl R, Nierhaus KH Assembly map of the large subunit (50S) of Escherichia coli ribosomes. Proc Natl Acad Sci U S A. 1982;79:729–33. doi:10.1073/pnas.79.3.729. 7038683 PMC345825

[35] Boore JL Animal mitochondrial genomes. Nucleic Acids Res. 1999;27:1767–80. doi:10.1093/nar/27.8.1767. 10101183 PMC148383

[36] Cheng R, Zheng X, Lin X, Yang J, Li Q Determination of the complete mitochondrial DNA sequence of Octopus minor. Mol Biol Rep. 2012;39:3461–70. doi:10.1007/s11033-011-1118-2. 21710247

[37] Gaitan-Espitia JD, Quintero-Galvis JF, Mesas A, D'Elia G Mitogenomics of southern hemisphere blue mussels (Bivalvia: Pteriomorphia): Insights into the evolutionary characteristics of the Mytilus edulis complex. Sci Rep. 2016;6:26853. doi:10.1038/srep26853. 27241855 PMC4886515

[38] Li Y, Wu X, Ji X, Yan P, Amato G The complete mitochondrial genome of salt-water crocodile (Crocodylus porosus) and phylogeny of crocodilians. J Genet Genomics. 2007;34:119–28. doi:10.1016/S1673-8527(07)60013-7. 17469784

[39] Wang ZL, Li C, Fang WY, Yu XP The Complete Mitochondrial Genome of two Tetragnatha Spiders (Araneae: Tetragnathidae): Severe Truncation of tRNAs and Novel Gene Rearrangements in Araneae. Int J Biol Sci. 2016;12:109–19. doi:10.7150/ijbs.12358. 26722222 PMC4679403

[40] Zhuang X, Qu M, Zhang X, Ding S A comprehensive description and evolutionary analysis of 22 grouper (perciformes, epinephelidae) mitochondrial genomes with emphasis on two novel genome organizations. PLoS One. 2013;8:e73561. doi:10.1371/journal.pone.0073561. 23951357 PMC3739747

